# Possible Interruption of Malaria Transmission, Highland Kenya, 2007–2008

**DOI:** 10.3201/eid1512.090627

**Published:** 2009-12

**Authors:** Chandy C. John, Melissa A. Riedesel, Ng’wena G. Magak, Kim A. Lindblade, David M. Menge, James S. Hodges, John M. Vulule, Willis Akhwale

**Affiliations:** University of Minnesota Medical School, Minneapolis, Minnesota, USA (C.C. John, M.A. Riedesel, D.M. Menge); Moi University School of Medicine, Eldoret, Kenya (N.G. Magak); Centers for Disease Control and Prevention, Atlanta, Georgia, USA (K.A. Lindblade); University of Minnesota, Minneapolis (J.S. Hodges); Kenya Medical Research Institute, Kisian, Kenya (J.M. Vulule); Ministry of Health, Nairobi, Kenya (W. Akhwale)

**Keywords:** Malaria, transmission, Plasmodium falciparum, epidemiology, Kenya, parasites, research

## Abstract

Highland areas where malaria transmission is unstable are targets for malaria elimination because transmission decreases to low levels during the dry season. In highland areas of Kipsamoite and Kapsisiywa, Kenya (population ≈7,400 persons), annual household indoor residual spraying with a synthetic pyrethroid was performed starting in 2005, and artemether/lumefantrine was implemented as first-line malaria treatment in October 2006. During April 2007–March 2008, no microscopy-confirmed cases of malaria occurred at the sites. In 4 assessments of asymptomatic persons during May 2007–April 2008, a total of <0.3% of persons were positive for asexual *Plasmodium falciparum* by microscopy or PCR at any time, and none were positive by PCR at the last 2 sample collections. Our findings show that in such areas, interruption and eventual elimination of malaria transmission may be achievable with widespread annual indoor residual spraying of households and artemisinin combination therapy.

## Abstract 2

Annual insecticide spraying and artemisinin combination therapy may stop transmission.

Widespread implementation of malaria control interventions such as insecticide-treated bed nets (ITNs) and artemisinin combination therapy (ACT) have resulted in dramatic reductions of transmission in areas where this disease is endemic (*1*–*3*). For the first time since the 1950s, the World Health Organization and other organizations are promoting malaria eradication (*4*). In highland areas (>1,500 m above sea level) in Africa, malaria transmission is unstable, with a low incidence of malaria during dry seasons (*5*). These areas may be ideal initial targets for attempting the interruption of malaria transmission. Also in these areas, the combination of ACT, which may decrease transmission by reducing gametocyte load in infected persons, and annual indoor residual spraying (IRS) with long-lasting insecticides, which can reduce indoor vector density for a prolonged period, could act synergistically to interrupt malaria transmission.

We have conducted malaria epidemiology studies in the adjoining highland areas of Kipsamoite and Kapsisiywa, Nandi Hills District, Kenya, since 2003. Starting in 2005, the Ministry of Health of Kenya introduced new interventions to reduce malaria transmission and improve malaria treatment in these areas. Interventions introduced included IRS, distribution of ITNs to pregnant women and their children <5 years of age, and provision of artemether/lumefantrine (co-artemether) as first-line therapy for uncomplicated malaria. The present study documents the changes in malaria transmission that occurred during the period of these interventions and provides evidence that malaria transmission was interrupted during April 2007–March 2008.

## Methods

### Study Site

The study was conducted in the adjacent highland areas of Kipsamoite (7 villages) and Kapsisiywa (9 villages) in the Nandi Hills District of Kenya. Study site characteristics have been described (*6*). Elevation ranges from 1,829 m to 2,132 m. In both areas, malaria transmission is unstable. A malaria epidemic occurred in Kipsamoite in 2002 (*7*). Historically, Kapsisiywa has had more malaria cases than Kipsamoite, likely because its elevation is lower, a swamp surrounds it, and it has more areas where water is likely to pool (*6*).

### Study Population and Recruitment

Persons living in these areas are predominantly Nandi, a Kalenjin subtribe. Demographic analysis, malaria surveillance, and collection of weather and vector density information were performed in these areas as part of 2 related studies, a study of malaria early warning systems and a study of malaria transmission and immunity. Written informed consent for study participation was obtained from consenting heads of households in the area for demographic studies and from persons (or parents/guardians of persons <15 years of age) for other studies. Ethical approval for the study was obtained from the Kenya Medical Research Institute National Ethical Review Committee and the Institutional Review Boards for Human Studies at Case Western Reserve University, the Centers for Disease Control and Prevention, and the University of Minnesota.

### Demography and Surveillance for Clinical Malaria and Asymptomatic Persons

Demographic surveys of all households were started in April 2003 and conducted every 4 to 6 months. Starting in 2005, these surveys included assessments of travel, ITN use, and IRS treatment of houses. Clinical malaria surveillance was conducted during 2003–2008 Kenyan Ministry of Health dispensaries, the only healthcare facilities within the study area. Free malaria diagnosis and treatment were available to all persons with symptoms of malaria. Persons with symptoms of malaria (fever, chills, severe malaise, headache) who did not have a clear alternative diagnosis by history and physical examination were assessed for malaria by microscopic examination of blood smears.

Clinical malaria was defined as symptoms of malaria and a positive blood smear for *Plasmodium falciparum* or *P*. *malariae*. Primary treatment for uncomplicated malaria was given according to Kenya Ministry of Health provisions for the clinics: sulfadoxine-pyrimethamine (2003–2004), amodiaquine (2004–2006), and co-artemether (2006–2008). Because of changes in studies, forms, and clinic personnel, clinical surveillance was not conducted in January and July–November 2006, in Kipsamoite; and July–December 2005, and January, March, and June–November 2006, in Kapsisiywa.

Four surveys for parasitemia in asymptomatic persons were conducted during 2007–2008. In May 2007, samples were collected from all consenting persons who were living on site during the time of collection (5,788 persons). In August 2007, samples were collected from 605 persons randomly selected from the overall population for active surveillance of malaria. Testing was repeated in samples from 577 and 538 of these persons in the cohort in November 2007 and April 2008, respectively. PCR was performed on 400 randomly selected samples from each period and on any sample that was parasite positive by microscopy.

### Microscopy and PCR

Microscopy testing for *Plasmodium* species was performed as described (*7*) by using 2 independent readings and a third reading for slides with discordant results. For PCR testing, genomic DNA was isolated with a QIAamp 96 DNA blood kit (QIAGEN Inc., Valencia, CA, USA) from dried blood spots collected on Whatman 903 filter paper (Whatman Corporation, Florham Park, NJ, USA). *P*. *falciparum* infection was detected by nested PCR specific for the small subunit RNA gene as described (*8*). In previous surveys in these areas, we documented low frequencies of *P*. *malariae* infection (<1%) and no *P*. *vivax* or *P*. *ovale* infections (*7*,*9*) by microscopy and PCR. Thus, we did not further test for these infections.

### Assessment of Rainfall, Temperature, and Indoor Resting Vector Density

Daily rainfall was measured with standard metal rain gauges, and daily minimum and maximum temperatures were recorded with maximum/minimum mercury thermometers placed in 7 villages in Kipsamoite. Values from the 7 villages were averaged.

Household indoor resting density of *Anopheles* spp. was measured in 4 cluster areas at each site; each cluster area comprised approximately equal numbers of persons. Three index households were randomly selected for each cluster, and the index house plus its 4 nearest neighbors were sampled. Sixty households in Kipsamoite and 60 households in Kapsisiywa were sampled every 2 weeks. Pyrethrum knockdown captures were conducted by using standard methods (*10*). *Anopheles* spp. mosquitoes were identified taxonomically, first in the field by trained field assistants and then by an entomologist from the Kenya Medical Research Institute.

### Data Analysis

Annual malaria incidence, rainfall, temperature, and vector density during April 2003–March 2004, were used as baseline data and compared with data from subsequent years beginning in April 2004. Annual malaria incidence was compared by using negative binomial regression analysis, except for April 2007–March 2008, when it was compared by Fisher exact test because no cases occurred during that year. For years with missing months of incidence data, malaria incidence in the recorded months was compared with incidence in the same months in the reference year (April 2003–March 2004). For periods when no cases were detected, a 1-sided 95% confidence interval was constructed. Mean daily temperature and mean monthly rainfall were compared by using the 2-sample *t* test. Median monthly vector density was compared by using the Wilcoxon rank-sum test. All data analysis was conducted by using Stata version 10.0 software (Stata Corp., College Station, TX, USA).

## Results

### IRS, ITNs, and Malaria Treatment with Co-artemether at Health Centers

The population ranged from 3,250 to 4,253 in Kipsamoite and from 3,412 to 3,841 in Kapsisiywa. The Ministry of Health first conducted limited indoor residual spraying in 2005. The number of households sprayed increased markedly in 2007 (Table 1). The primary insecticide used in both sites was lambda cyhalothrin at a dose of 10–20 mg/m^2^.

**Table 1:** Indoor residual spraying of households in Kipsamoite and Kapsisiywa, Kenya, 2005–2007

Year, area	Months of spraying	No. households sprayed/total (%)
2005		
Kipsamoite	Apr–May	37/770 (4.8)
Kapsisiywa	Apr–Jun	374/713 (52.5)
2006		
Kipsamoite	Feb–May	119/786 (15.1)
Kapsisiywa	Feb–May	327/716 (45.70
2007		
Kipsamoite	Apr–Jul	545/773 (70.5)
Kapsisiywa	Apr–Jun	656/690 (95.1)

ITNs were distributed per Ministry of Health policy to pregnant women and their children <5 years of age when the women came to the health centers for antenatal care. A copayment was required in most instances. Short-lasting nets treated with α-cypermethrin were provided during 2005–2006, and long-lasting nets treated with deltamethrin or permethrin have been provided since 2006. Persons were asked, “Have you slept under a treated bednet regularly since the last demography survey?” During 2006–2008, the percentage of persons who reported sleeping under an ITN decreased from 17.3% to 11.1% in Kipsamoite and from 27.9% to 15.1% in Kapsisiywa.

Co-artemether became official first-line treatment for uncomplicated malaria by Kenyan Ministry of Health policy in May 2004, but it was not implemented in these sites until October 23, 2006 (Kapsisiwya) and February 6, 2007 (Kipsamoite). Co-artemether was also not widely available in local shops or neighboring clinics before these dates.

### Malaria and Symptoms Consistent with Malaria, 2003–2008

**Figure 1:**
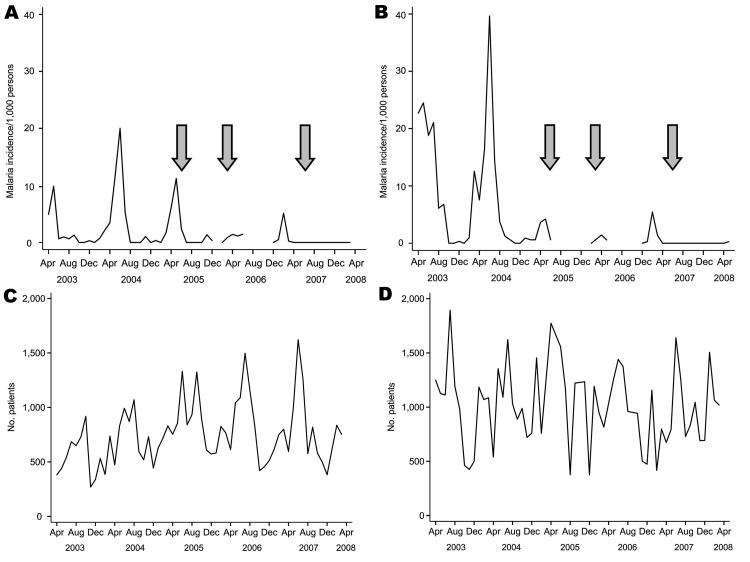
Malaria incidence and number of patients seen at health dispensaries in 2 highland areas of western Kenya, April 2003–March 2008. A) Monthly incidence of malaria/1,000 persons in Kipsamoite. B) Monthly incidence of malaria/1,000 persons in Kapsisiywa. C) No. patients who came to the Kipsamoite health dispensary. D) No. patients who came to the Kapsisiywa health dispensary. Gaps in panels A and B indicate that no data were collected during these periods. Arrows indicate when indoor residual spraying was conducted in the 2 areas.

When compared with the April 2003–March 2004 reference year, malaria incidence in Kipsamoite increased in 2004, returned to 2003 levels in 2005, and decreased in 2006 (Table 2, Figure 1, panel A). In contrast, malaria incidence decreased slightly in Kapsisiywa in 2004 and then decreased in 2005 and 2006 (Table 2, Figure 1, panel B). Malaria incidence in Kapsisiywa was higher than in Kipsamoite in 2003 (p<0.001) and 2004 (p = 0.06), but not in any other year. At both sites during March 20, 2007–March 30, 2008, microscopically confirmed cases of malaria were found among symptomatic persons (Table 2, Figure 1, panels A and B).

**Table 2:** Annual cumulative incidence of *Plasmodium falciparum* malaria in Kipsamoite and Kapsisiwya, Kenya, April 2003–March 2008*†

Year	Kipsamoite				Kapsisiywa		
	Cumulative incidence	Incidence ratio (95% CI)	p value		Cumulative incidence	Incidence ratio (95% CI)†	p value
2003 Apr–2004 Mar	23.20	Ref	Ref		106.03	Ref	Ref
2004 Apr–2005 Mar	42.53	1.83 (1.36–2.47)	<0.001		82.58	0.78 (0.66–0.91)	0.002
2005 Apr–2006 Mar	18.79‡	0.81 (0.57–1.16)§	0.229		8.03¶	0.10 (0.07–0.15)#	<0.001
2006 Apr–2007 Mar	9.30**	0.47 (0.30–0.71)††	<0.001		8.99‡‡	0.19 (0.12–0.27)§§	<0.001
2007 Apr–2008 Mar	0.00	0.00	<0.001		0.00	0.00	<0.001

*CI, confidence interval; Ref, reference. †Annual cumulative incidence/1,000 persons was compared by using negative binomial regression, except for 2007–2008, which was compared by using Fisher exact test. ‡Available data were for all months except January 2006. §Compared with same months in 2003–2004 (incidence 23.20/1,000). ¶Available data were for April–June 2005 and February 2006. #Compared with same months in 2003–2004 (incidence 79.41/1,000). **Available data were for April–June 2006 and December 2006–March 2007. ††Compared with same months in 2003–2004 (incidence 19.99/1,000). ‡‡Available data were for April–May 2006 and December 2006–March 2007. §§Compared with same months in 2003–2004 (incidence 48.19/1,000).

Overall clinic attendance, although seasonal and variable, did not decrease during 2007–2008 at either site (Figure 1, panels C and D). During March 20, 2007–March 30, 2008, when 0 of 416 symptomatic persons had evidence of *P*. *falciparum* parasitemia by microscopy (0%, 1-sided 95% confidence interval 0%–0.9%), 17 (7.3%) of 231 symptomatic persons who provided blood samples for PCR testing were positive for *P*. *falciparum*. All PCR-positive samples were obtained during March–June, 2007, except for 2 samples, which were obtained in December 2007. In symptomatic persons, gametocyte prevalence, assessed by microscopy, was low in all years (2.8%, 2.9%, 0.8%, 0.8%, and 0% in Kipsamoite and 0.4%, 1%, 0%, 0%, and 0% in Kapsisiywa during 2003, 2004, 2005, 2006, and 2007, respectively).

### Asexual Parasitemia and Gametocytemia in Asymptomatic Persons, 2007–2008

In 4 surveys of asymptomatic persons during 2007–2008, a total of <0.3% were positive for *P*. *falciparum* trophozoites or gametocytes by microscopy during any period. In the last 2 periods, no person was positive for asexual *P*. *falciparum* by PCR (Table 3). The person positive for *P*. *falciparum* trophozoites by microscopy in April 2008 was also positive for gametocytes. PCR showed that this person did not have an asexual *P*. *falciparum* infection.

**Table 3:** Prevalence of *Plasmodium falciparum* parasitemia by microscopy and PCR in asymptomatic persons in Kipsamoite and Kapsisiywa, Kenya, May 2007–April 2008

Characteristic	2007 May	2007 Aug	2007 Nov	2008 Apr
Microscopy				
No. *P. falciparum* positive	14*	0	0	1
No. tested	5,788	605	577	538
% *P. falciparum* positive	0.24	0	0	0.19
Gametocytes				
No. positive	5†	0	0	1
No. tested	5,788	605	577	538
% positive	0.09	0	0	0.19
PCR				
No. *P. falciparum* positive	1	1	0	0
No. tested	414	400	400	401
% *P. falciparum* positive	0.24	0.25	0	0

*Twelve persons who tested positive by microscopy were from Kipsamoite and 2 were from Kapsisiywa. †Four persons who tested positive by gametocyte were from Kipsamoite and 1 was from Kapsisiywa.

### Changes in Rainfall, Temperature, and Vector Density, 2003–2008

**Figure 2:**
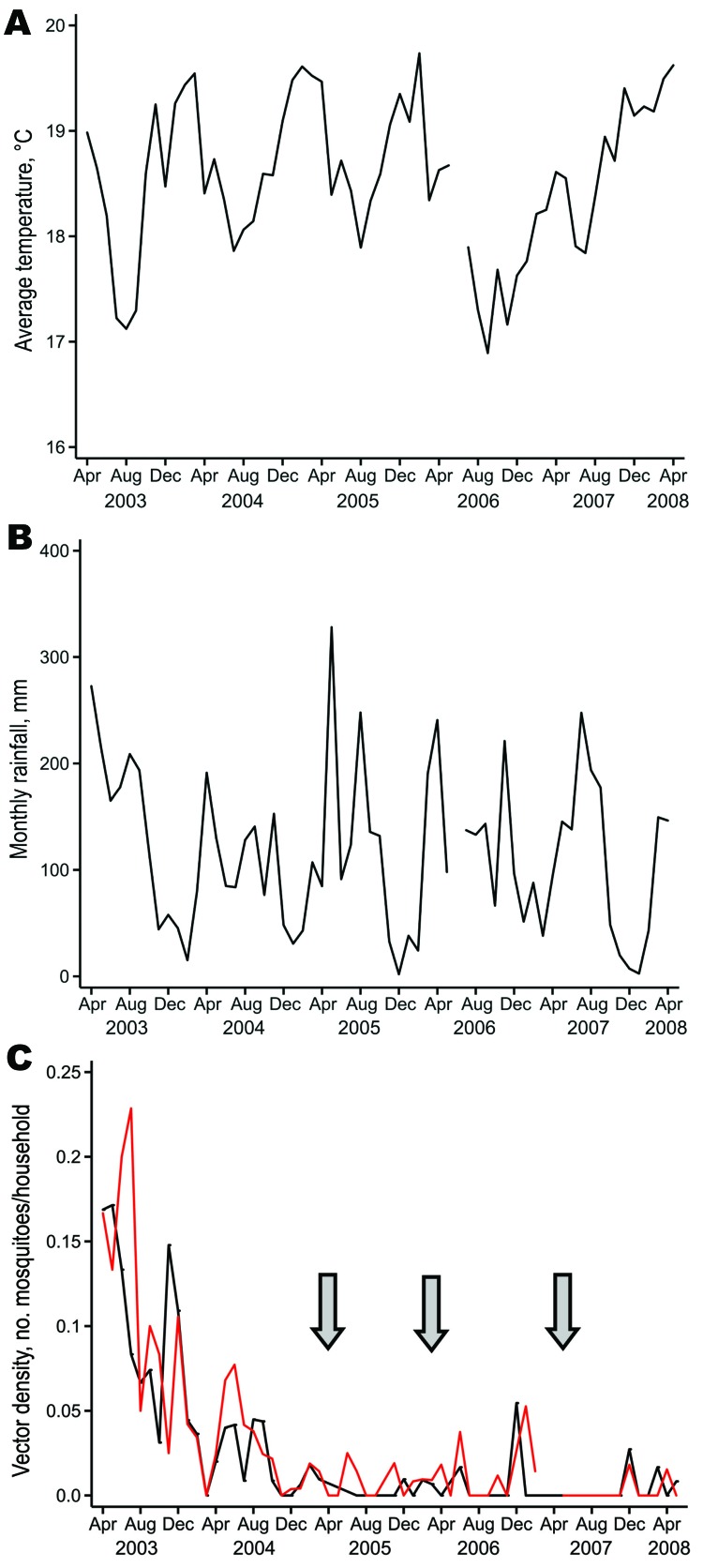
Temperature, rainfall, and vector density in 2 highland areas of western Kenya, April 2003–May 2008. A) Average daily temperature (°C) in Kipsamoite. B) Monthly rainfall (mm) in Kipsamoite. C) Median biweekly vector density (no. *Anopheles* spp. mosquitoes/household) in Kapsisiywa (red line) and Kipsamoite (black line). Gaps in panels indicate that no data were collected during these periods. Arrows indicate times when indoor residual spraying was conducted.

During 2003–2008, no consistent pattern of increased or decreased temperature was shown, and the average daily temperature during 2007–2008, the year in which no malaria cases were documented by microscopy, was most similar to that in 2003, the year of highest malaria incidence (Figure 2, panel A, Table 4). Average monthly rainfall did not differ between years (Figure 2, panel B, Table 4). A decrease in anopheline mosquito density was seen in 2004, before any widespread IRS treatment program, but sustained low levels of mosquito density were first seen after IRS treatments (Figure 2, panel C; Table 5). Of 447 anopheline mosquitoes caught over this 5-year period, 439 (98.21%) were identified as *An*. *gambiae*, 6 (1.34%) were identified as *An*. *funestus*, and 2 (0.45%) were not identified.

**Table 4:** Average monthly rainfall and average daily temperature for Kipsamoite, Kenya, April 2003–March 2008*

Year	Mean (SD) monthly total rainfall, mm†	p value	Mean (SD) daily temperature, °C†	p value
2003 Apr–2004 Mar	132.8 (83.6)	Ref	18.5 (0.9)	Ref
2004 Apr–2005 Mar	101.4 (48.9)	0.274	18.7 (0.6)	0.510
2005 Apr–2006 Mar	119.2 (97.6)	0.717	18.8 (0.6)	0.346
2006 Apr–2007 Mar	130.5 (76.7)	0.957	17.8 (0.6)	0.039
2007 Apr–2008 Mar	105.2 (81.6)	0.422	18.8 (0.6)	0.352

*Ref, reference. †Means were compared by using the 2-sample *t* test.

**Table 5:** Median monthly *Anopheles* spp. mosquitoes per household in Kipsamoite and Kapsisiwya, Kenya, April 2003–March 2008*

Year	Kipsamoite			Kapsisiywa	
	Median monthly *Anopheles* spp. mosquito density (IQR)†	p value		Median monthly *Anopheles* spp. mosquito density (IQR)†	p value
2003 Apr–2004 Mar	0.079 (0.100)	Ref		0.091 (0.112)	Ref
2004 Apr–2005 Mar	0.014 (0.033)	0.003		0.023 (0.031)	0.005
2005 Apr–2006 Mar	0.000 (0.007)	<0.001		0.009 (0.012)	<0.001
2006 Apr–2007 Mar	0.000 (0.004)	<0.001		0.006 (0.018)	0.002
2007 Apr–2008 Mar	0.000 (0.000)	<0.001		0.000 (0.000)	0.002

*IQR, interquartile range (difference between 75th and 25th percentile values); ref, reference. †Median vector density was compared by using the Wilcoxon rank-sum test.

## Discussion

Implementation of highly effective methods of decreasing malaria transmission, including ITNs, IRS, and ACT, has led to renewed discussion about global eradication of malaria (*4*,*11*). A major goal in moving toward eradicating malaria is interruption or elimination of local malaria transmission. The World Health Organization has stated that “elimination has been achieved when the ‘prevention of reintroduction’, without local transmission by mosquitoes, has been successful for three or more consecutive years” (*12*). Interruption of local transmission is the step before elimination, in which it is documented that local transmission of malaria is absent in a previously malaria-endemic area for a specific period. The present study provides microscopy evidence of interruption of local malaria transmission in 2 adjacent highland areas of unstable transmission. Malaria could recur in these areas, and it is unclear precisely how much specific factors (e.g., IRS, ACT, and changes in rainfall and temperature) affected malaria transmission and incidence. Overall, however, data support the idea that in unstable transmission settings, combining regular, widespread IRS campaigns and use of ACT as first-line antimalarial treatment has the potential to interrupt local malaria transmission.

IRS probably played a major role in reducing malaria transmission in these areas for several reasons. First, sustained decreases in indoor resting *Anopheles* spp. mosquito density were seen after the IRS campaigns. Second, in both sites, malaria incidence decreased only after IRS was widely applied. In Kipsamoite, no large reduction in malaria incidence was seen until 2007, when spraying covered >70% of households. In Kapsisiywa, ≈50% of households were sprayed in 2005 and 2006, and a large decrease in malaria incidence was observed in both years. In 2007, after >90% of households were sprayed, malaria transmission was interrupted for the subsequent year. Third, in contrast to IRS, 2 factors that could affect vector density and therefore malaria incidence, rainfall and temperature, showed no clear relationship with either vector density or malaria incidence.

A reduction in vector density was seen in both sites in 2003 before the IRS campaigns. Potential reasons for this reduction include an unusual decrease in temperature during July–September 2003 (Figure 2). This decrease in temperature coincided with the first decrease in vector density; another possible reason for the decrease was pyrethrum spray catch testing of anopheline vectors conducted by our team, which started in April 2003. Although spraying with short-term insecticide does not usually affect vector density in areas of high transmission, spraying of approximately one sixth of all households every 2 weeks may have had an effect on the adult vector population, which led to a smaller breeding pool and lower overall vector density in this area of low transmission. The decrease in malaria incidence in Kipsamoite in 2005, after only 15% of households were sprayed, may in part reflect the effects of greater spraying in neighboring Kapsisiywa. The decrease may also reflect the combined effect of partial coverage with ITNs and additional coverage by IRS. Reduction of incidence in Kipsamoite in 2005 was not caused by concentrated spraying in areas of malaria clustering (*5*) because spraying was nearly absent in these areas. Reductions in malaria incidence were seen in Kapsisiywa in 2005–2006 after spraying of 40%–50% of households, but the small peak in incidence seen subsequently in these 2 years, but not 2007 (Figure 1), suggests that for interruption, a higher percentage of households (>70%) must be sprayed.

Treatment of malaria patients with co-artemether reduces gametocyte carriage and density in children and makes them less infectious to mosquitoes than treatment with sulfadoxine-pyrimethamine plus chloroquine (*13*). The effect of co-artemether on gametocytes may have been synergistic with the effect of widespread IRS on the *Anopheles* spp. vector in reducing malaria transmission. In symptomatic persons, gametocyte prevalence was always low, and it decreased before introduction of co-artemether, but it did not decrease to undetectable levels in Kipsamoite until after introduction of co-artemether. Among asymptomatic persons, studies during 1999–2002 generally demonstrated higher gametocyte prevalence (0%–5.7%) (*7*). Lower prevalence among asymptomatic persons in the current study (0%–0.2% during May 2007–April 2008) could reflect effects of co-artemether on gametocyte prevalence after introduction of co-artemether in late 2006–early 2007, but without interim data from 2002–2006, an association cannot be clearly demonstrated. As with IRS, however, absence of microscopy-positive malaria cases occurred only after introduction of co-artemether. Because co-artemether was first used during a time of low transmission of malaria, the contribution of ACT to the absence of malaria incidence could not be quantified in the present study. However, a much larger study in South Africa in which IRS treatment and ACT treatment of persons with clinical malaria were introduced sequentially demonstrated an additional reduction of malaria incidence after introduction of ACT (*14*) and this supports the idea of synergy between these 2 interventions.

Although ITNs are the preferred intervention for preventing malaria-related illness and death in areas of high transmission (*1*,*15*,*16*), ITNs probably did not play a major role in interrupting malaria transmission in the highland areas we studied. ITN coverage never exceeded 30% in either area, and use actually decreased over the study period. In areas of unstable transmission, IRS treatment once a year is likely to be easier, more effective, and more accepted than ITNs. The Roll Back Malaria program currently recommends IRS as the preferred method of reducing malaria in areas of low transmission (*17*); our study supports this recommendation.

Insecticide treatment using IRS is not without problems; chief among them is potential development of resistance to the insecticide. Lambda-cyhalothrin, the insecticide used for IRS in these areas by the Kenyan Ministry of Health, was used in Mozambique for IRS starting in 1993, but resistance developed to such an extent that lambda cyhalothrin was replaced by bendiocarb in 2000. By 2006, however, lambda cyhalothrin resistance had decreased in many areas (*18*). In a recent study in nearby areas of western Kenya, no phenotypic resistance to pyrethroid insecticides was seen, but 27% of anophelines carried the knockdown resistance (*kdr*) mutation associated with increased resistance to pyrethroids (*19*). Assessment for insecticide resistance in the highland areas of the present study will enable better policy decisions to be made about continued use of lambda cyhalothrin, use of alternatives such as bendiocarb or DDT, or cycling of insecticides when certain resistance thresholds are reached for a particular insecticide. *P*. *falciparum* resistance to sulfadoxine-pyrimethamine was present in 27% of infections in western Kenya as early as 1999 (*20*). Resistance to co-artemether has not yet been documented in Kenya, but development of resistance to components of artemether/lumefantrine in nearby populations with higher levels of transmission would also pose a threat to this highland population. Monitoring of drug resistance to ACT in all areas in which malaria is endemic will be critical for sustaining reduction of malaria incidence in sub-Saharan Africa.

Limitations of our study include missing monthly malaria incidence data among symptomatic persons during 2005–2006, the observational nature of the study, and the possibility that lack of parasitemia by microscopy and PCR in symptomatic and asymptomatic persons was caused by seasonal variation common in highland areas (*21*) and not by interruption of local transmission. However, 3 pieces of evidence from studies of asymptomatic and symptomatic persons support interruption of local transmission rather than seasonal variation. First, no parasitemia was seen in 2 successive microscopy and PCR surveys of asymptomatic persons, whereas in 5 earlier surveys of asymptomatic persons in Kipsamoite, the area of lower transmission, the frequency of infected persons was never zero (range 5.9%–14.5% by PCR) (*7*). Second, over 7 years of clinic surveillance of symptomatic persons in Kipsamoite and 5 years in Kapsisiywa, there was never a >4-month period in which there were no microscopy-positive cases of *P*. *falciparum* before March 2007. Thus, even with seasonal variation, a year with no microscopy-positive cases in these areas is unprecedented. Third, absence of gametocytemia by microscopy was documented among asymptomatic persons in 2 of 4 assessments and among symptomatic persons for a year (April 2007–March 2008), which suggested that the potential for local transmission was low or absent.

Because reverse transcription–PCR methods for detection of gametocytes have documented higher rates of gametocyte infection than microscopy (*22*,*23*), we are developing this testing method in our laboratory to confirm the absence of gametocytemia in the most recent samples from study participants. The presence of asexual *P*. *falciparum* infection by PCR in 15 symptomatic persons during April–June 2007 could reflect prolonged detection by this more sensitive method. The presence of only 2 PCR-positive cases in a 9-month period (July 2007–March 2008) suggests that malaria transmission was either interrupted, if these cases were caused by patients’ travel, or reduced to almost undetectable levels.

Sustained elimination of local malaria transmission in these areas will require ongoing surveillance of malaria incidence, anopheline vector density, and anopheline insecticide resistance, and correctly timed IRS campaigns with broad coverage of the area (*24*). The longer populations at these sites are unexposed to malaria, the more susceptible they are to malaria epidemics, which could occur if an increase in vector density occurs in conjunction with the arrival of infected persons or mosquitoes from an area of higher transmission of malaria. Because travel is increasingly frequent, true elimination of malaria in this and other highland areas will require reduction and eventual elimination of malaria in surrounding areas. Co-artemether must be consistently available to treat any infected and symptomatic travelers or immigrants to the area. Finally, as malaria cases decrease, microscopists will need to receive training to remain proficient in detection of malaria in blood smears.

In summary, this study demonstrates pronounced reduction and possible interruption of malaria transmission in 2 highland areas of Kenya for a 1-year period and provides evidence that interruption of transmission was related to widespread annual IRS insecticide treatment and use of ACT as first-line treatment for uncomplicated malaria. Although both areas remain at risk for recurrence of malaria epidemics, our study provides evidence that interruption and eventual elimination of malaria in areas of unstable transmission may be achievable.
